# Operator Influence on Blinded Diagnostic Accuracy of Point-of-Care Antigen Testing for Group A Streptococcal Pharyngitis

**DOI:** 10.1155/2016/1710561

**Published:** 2016-08-04

**Authors:** Carla Penney, Robert Porter, Mary O'Brien, Peter Daley

**Affiliations:** ^1^Faculty of Medicine, Memorial University of Newfoundland, St. John's, NL, Canada A1B 3V6; ^2^Newfoundland and Labrador Eastern Health, St. John's, NL, Canada A1B 3V6

## Abstract

*Background*. Acute pharyngitis caused by Group A* Streptococcus* (GAS) is a common presentation to pediatric emergency departments (ED). Diagnosis with conventional throat culture requires 18–24 hours, which prevents point-of-care treatment decisions. Rapid antigen detection tests (RADT) are faster, but previous reports demonstrate significant operator influence on performance.* Objective*. To measure operator influence on the diagnostic accuracy of a RADT when performed by pediatric ED nurses and clinical microbiology laboratory technologists, using conventional culture as the reference standard.* Methods*. Children presenting to a pediatric ED with suspected acute pharyngitis were recruited. Three pharyngeal swabs were collected at once. One swab was used to perform the RADT in the ED, and two were sent to the clinical microbiology laboratory for RADT and conventional culture testing.* Results*. The RADT when performed by technologists compared to nurses had a 5.1% increased sensitivity (81.4% versus 76.3%) (*p* = 0.791) (95% CI for difference between technologists and nurses = −11% to +21%) but similar specificity (97.7% versus 96.6%).* Conclusion*. The performance of the RADT was similar between technologists and ED nurses, although adequate power was not achieved. RADT may be employed in the ED without clinically significant loss of sensitivity.

## 1. Introduction 

Acute pharyngitis is a common presentation to primary care physicians and pediatric ED, accounting for 6–8% of visits each year in high-income nations [1, 2]. While most cases of acute pharyngitis are viral in origin, 20–40% [1, 3] of cases are caused by Group A* Streptococcus* (GAS) infection. 60–70% of children presenting with acute pharyngitis will be prescribed an antibiotic [1, 4], suggesting that appropriate diagnostic testing is not always performed, and antimicrobial stewardship could be improved. Considering the high prevalence, stewardship impact could be significant.

Differentiating between viral and GAS pharyngitis is difficult, with even the most experienced clinician being unable to discern the signs and symptoms reliably [3]. Clinical prediction rules (e.g., Centor criteria [5] and McIsaac score [6]) have been developed to aid clinicians in predicting GAS infection, but the performance of rules is not high enough to inform treatment without culture [2, 3, 7]. The reference standard for diagnosing GAS pharyngitis is a throat swab cultured on selective agar. Culture has a sensitivity of approximately 90% to 95% and specificity of approximately 99% [8] but requires 18–24 hours incubation, which prevents point-of-care treatment decisions and requires a second contact with the patient to provide results.

Rapid antigen-detecting tests (RADT) for diagnosis of GAS demonstrate excellent specificity (approximately 95%) but variable sensitivity (66%–99%) [3, 9]. Sensitivity is influenced by disease severity, size of the bacterial inoculum obtained on the swab, and operator influence on testing technique [3]. When nursing staff and laboratory technicians perform the same RADT, diagnostic performance of technologists is significantly better, with a difference in sensitivity ranging from 14% to 34% between groups [10, 11]. This may be due to operator experience, compliance with the method when performing the test, experience in reading RADTs, or other unidentified reasons [10]. This operator influence may reduce clinical utility. The RADT is specifically designed for simplicity of testing, such that operator influence should be minimized.

The objective of this study was to measure operator influence on the diagnostic accuracy of a RADT when performed by trained pediatric ED nurses and clinical microbiology laboratory technologists, with conventional culture as the reference standard.

## 2. Methods

Prior to initiation of the study, ED nurses were trained in person and provided a training video and poster explaining the principle of the study and how to perform the RADT; approximately 30 nurses were trained. ED physicians were provided the same training video as some sections of the video pertained to them (i.e., how to collect a proper throat swab) (available at the following URL: https://www.youtube.com/watch?v=_1UjwYlbgCo). Physicians performed the swab collection and nurses performed the RADT. Laboratory staff were provided the package insert, without training.

Ethics and institutional approvals were obtained from the local research ethics board prior to study initiation. From November 2015 to January 2016, consecutive children presenting to the Janeway Children's Hospital ED in St. John's, NL, Canada, with suspected pharyngitis were recruited into the study by parental consent. The sole exclusion criterion was current antibiotic treatment. During triage assessment, the child was determined by the triage nurse to have possible pharyngitis (based on history without physical examination), and consent for participation was obtained from the parent or guardian. The ER physician would then assess the child and perform a physical examination. If pharyngitis was suspected, the physician would perform a single triplicate pharyngeal swab collection using three Copan eSwabs (Copan Diagnostics Inc., California, USA) held together. One swab was used to perform the RADT in the ED, and two swabs were sent to the microbiology laboratory for the technologists to perform the RADT and conventional culture. The physicians made independent treatment decisions.

The RADT evaluated was Alere™ TestPack Plus Strep A kit (Alere ULC, Ontario, Canada), which is a rapid immunochromatographic assay. The kit contains three extraction reagents, and a reaction disc to which the extraction solution was added. The reaction disc has two internal controls. The test was performed according to the manufacturer's specifications. The test was performed on the date of collection.

Conventional culture was performed according to laboratory protocol, using* Streptococcus* selective agar, with beta-hemolytic colonies confirmed by using latex agglutination. Groups C and G* Streptococcus* were not reported. The test was performed on the date of collection.

Sensitivity and specificity were defined as a comparison of RADT with culture. With an expected reduction in sensitivity from 80% sensitivity for technologist-performed RADT to 65% sensitivity for nurse-performed RADT (a reduction in sensitivity of 15% [10], type I error risk of 0.05 and a power of 80%), using a two-sided test, a sample size of 140 specimens was calculated. Confidence intervals were determined using an online statistical calculator (MedCalc Software v15.8, Ostend, Belgium) (https://www.medcalc.org/calc/diagnostic_test.php). Comparison between performance was calculated using McNemar's test. Missing or indeterminate results were not included in analysis. Analysis was performed using SPSS 20.0 (IBM, USA). A two-sided *p* value of <0.05 was considered statistically significant.

## 3. Results 

**Figure 1:**
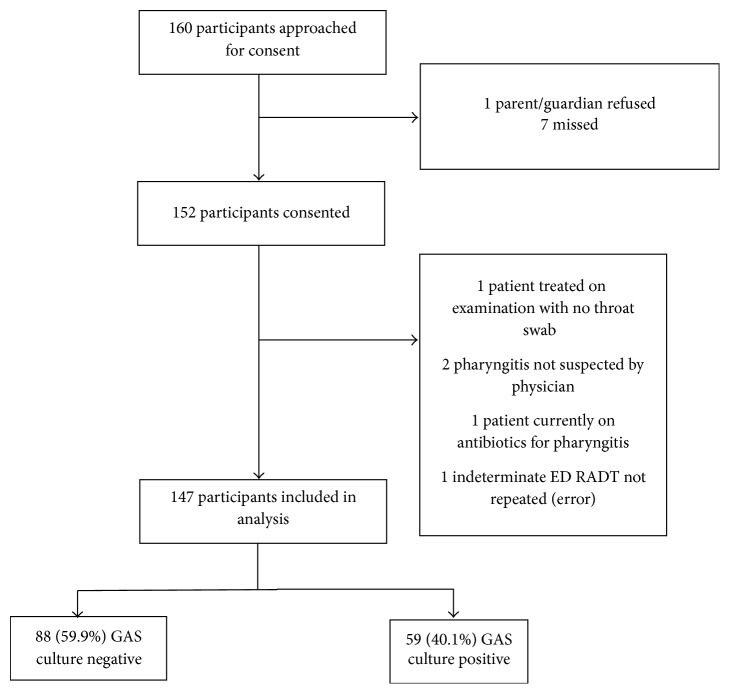
Participant flow.

Of the 160 participants approached for consent, 147 were included for analysis (Figure 1). Participant mean age was 8.8 ± 4.3 years, and 53.1% were females.

Culture detected 59/147 = 40.1%, nurse-performed RADT detected 45/147 = 30.6%, and technologist-performed RADT detected 48/147 = 32.7%. The difference between nurse-performed RADT detection rate and technologist-performed RADT detection rate was −2.1% (95% CI = −8.96, 13.11).  Table 1 outlines the sensitivity and specificity of the RADT compared to culture. Technologist-performed RADT demonstrated a 5.1% increased sensitivity (95% CI for difference between technologists and nurses = −11% to +21%) compared to nurse-performed RADT (81.4% versus 76.3%) (Table 1). Nurses reported three more false negative tests and one more false positive test than technologists (Table 2). Specificity was similar (97.7% versus 96.6%). The sensitivity difference was not statistically significant (*p* = 0.791).

## 4. Discussion 

We evaluated the operator influence on performance of RADT in the pediatric ED setting and found a nonsignificant difference between nurses and technologists. GAS prevalence was comparable to similar studies which had GAS detection rates ranging from 22% to 38% [4, 9, 12, 13]. We observed a smaller operator effect than predicted from previous literature [10, 11], and therefore our study was underpowered to detect a significant difference, despite achieving our calculated sample size. Our sample size was calculated using the expected difference in sensitivity between technologist and nurse-performed RADT. We calculated the sample size as total number of specimens; however, the correct calculation should have been total number of* positive* specimens; therefore, our sample size was too low to reach the conclusion statistically.

While an absolute difference in sensitivity of 5.1% was observed, the confidence limits for this difference range from −11% to +21%, demonstrating that technologist-performed RADT may be up to 21% more sensitive than nurse-performed RADT. A five percent difference in sensitivity would create a 2.1% increase in detection rate, if all RADTs were performed by laboratory technologists. This small difference in sensitivity may be interpreted as clinically insignificant and may be overwhelmed by the workflow benefits favouring RADT use in ED.

The explanation for a smaller operator influence in our study may be the extensive training received by nurses or the Hawthorne effect due to participation in a study. What it does demonstrate is that point-of-care RADT performance may approach lab RADT performance in an ideal setting.

Fox et al. found that the sensitivity of RADT when performed by laboratory technologists was significantly higher (*p* < 0.0001) than nonlaboratory personnel [11] (88% versus 56%). A blinded evaluation of performance using external quality assurance samples found a significant operator difference among both strongly positive specimens (correct results 98.9% versus 95.1% *p* < 0.001) and weakly positive specimens (79.3% versus 65.3% *p* < 0.001), suggesting that operator influence was larger among weak positives [10]. RADTs evaluated in these studies were different than the RADT evaluated in the present study, although based on the same detection method (immunochromatographic assay).

The main explanation for operator influence is experience [11]. Laboratory technologists are trained to perform testing precisely, but nurses may not perform tests exactly according to the manufacturer's specifications (e.g., adding an extra drop of reagent) [10]. Nurses without experience in point-of-care testing may be insecure in deciding which lines to interpret as positive. Furthermore, technologists acquire more experience through a higher volume of testing.

Sensitivity of RADTs may be influenced by disease severity (spectrum bias) [12, 13] and the quality of the specimen obtained from the pharynx [12]. Furthermore, the use of a throat culture as a reference standard may be inadequate since at most a throat culture will detect only 90–95% of GAS in symptomatic patients [10] and is unable to differentiate between colonization and active infection. PCR testing may be a more reliable reference standard when comparing performance of RADTs and their operators [9].

Our study had some limitations. We were underpowered to make a statistical inference between operators. While proper technique was demonstrated in obtaining a throat swab, collection technique was not standardized, which could influence results. Lastly, the study was short in duration. Had it been extended, we may have observed less operator influence as nurses acquired experience. We did not monitor changes in operator effect over time during the study period.

## Figures and Tables

**Table 1:** Performance comparison.

	Nurse-performed RADT	Technologist-performed RADT
Sensitivity (%) (95% C.I.)	76.3 (63.4, 86.4)	81.4 (69.1, 90.3)
Specificity (%) (95% C.I.)	96.6 (90.4, 99.3)	97.7 (92.0, 99.7)
Negative predictive value (%) (95% C.I.)	85.9 (77.4, 92.1)	88.7 (80.6, 94.2)
Positive predictive value (%) (95% C.I.)	93.8 (82.8, 98.7)	96.0 (86.3, 99.5)

**Table 2:** Comparison of technologist-performed RADT and nurse-performed RADT.

	Culture positive (*N* = 59)	Culture negative (*N* = 88)
Technologist and nurse RADT positive	47	0
Technologist RADT positive/nurse RADT negative	0	2
Technologist RADT negative/nurse RADT positive	1	0
Technologist and nurse RADT negative	11	86
